# Gliadel wafers in the treatment of malignant glioma: a systematic review

**DOI:** 10.3747/co.2007.147

**Published:** 2007-10

**Authors:** J. Perry, A. Chambers, K. Spithoff, N. Laperriere

**Affiliations:** * Toronto–Sunnybrook Regional Cancer Centre, Toronto, Ontario; † Cancer Care Ontario, Program in Evidence-Based Care, McMaster University, Hamilton, Ontario.; ‡ Princess Margaret Hospital, Toronto, Ontario

**Keywords:** Gliadel, interstitial chemotherapy, carmustine, malignant glioma, glioblastoma, systematic review

## Abstract

**Question:**

What is the safety and efficacy of interstitial chemotherapy with carmustine-loaded polymers (Gliadel wafers: MGI Pharma, Bloomington, MN, U.S.A.) in the treatment of newly diagnosed or recurrent malignant glioma (that is, glioblastoma multiforme, anaplastic astrocytoma, anaplastic oligoastrocytoma, and anaplastic oligodendroglioma)?

**Perspectives:**

Malignant glioma is the most common type of primary brain tumour in adults. In general, efficacy of systemic therapy in this patient population has been disappointing, and novel treatment approaches are needed. Because several randomized controlled trials (rcts) investigating the safety and efficacy of Gliadel are available, the Neuro-oncology Disease Site Group of Cancer Care Ontario’s Program in Evidence-Based Care decided that a systematic review of the evidence was necessary.

**Outcomes:**

The outcomes of interest for this review were overall survival, adverse events, and quality of life.

**Methodology:**

Systematic searches of the medline, embase, and Cochrane Library databases were conducted for relevant evidence. Fully-published reports of rcts comparing treatment with Gliadel wafers to placebo or alternative treatment were selected for inclusion. Prospective cohort studies were also included.

**Results:**

Two rcts that compared Gliadel to placebo in patients with newly diagnosed malignant glioma were obtained. Both rcts reported a significant survival benefit for patients who received Gliadel as compared with patients in the control group. One rct and one prospective cohort study were obtained that examined the role of Gliadel in patients with recurrent malignant glioma. The rct demonstrated a significant survival benefit for Gliadel only after adjustment for prognostic factors, and the prospective cohort study reported no survival benefit for Gliadel as compared with a historical control group. All three rcts reported similar rates of adverse events in the treatment and control groups. The most frequently reported adverse events were convulsions, confusion, brain edema, infection, hemiparesis, aphasia, and visual field defects.

**Conclusions:**

Gliadel is an option for selected patients with newly diagnosed malignant glioma where a near gross total resection is possible. No evidence is available comparing Gliadel with systemic therapy, and a decision to combine Gliadel with systemic therapy should be made for patients individually. The patient population that would benefit from Gliadel (age, histology, and performance status) is unclear; further investigation is needed. Gliadel is also an option for patients with surgically resectable recurrent malignant glioma.

## 1. QUESTION

What is the safety and efficacy of interstitial chemotherapy with carmustine-loaded polymers (Gliadel wafers: MGI Pharma, Bloomington, MN, U.S.A.) in the treatment of newly diagnosed or recurrent malignant glioma [that is, glioblastoma multiforme (gbm), anaplastic astrocytoma, anaplastic oligoastrocytoma, and anaplastic oligodendroglioma]?

Outcomes of interest for this guideline were overall survival, adverse events, and quality of life.

## 2. CHOICE OF TOPIC AND RATIONALE

Malignant glioma is the most common type of primary brain tumour in adults. Approximately 5 new cases per 100,000 population are diagnosed each year.

The current standard treatment for malignant glioma consists of surgical resection followed by radiation therapy. On recurrence, regimens of systemic chemotherapy delivered by the intravenous or oral route are used. Median survival remains poor despite refinement in surgical techniques and radiation therapy delivery.

Nitrosoureas, especially carmustine (bcnu) and, more recently, temozolomide, are the agents most frequently used in systemic chemotherapy. Temozolomide concurrently with radiotherapy and as adjuvant therapy has shown promising survival benefits with low toxicity, but the clinical effectiveness of systemic therapy in general has been disappointing. Systemic toxicities, short half-life, and limitations in traversing the blood–brain barrier are common problems limiting the clinical effectiveness of systemic agents. Novel methods for treating malignant gliomas are needed and should be evaluated to assess their role in this devastating disease.

Gliadel wafers represent a novel approach to the delivery of chemotherapy in malignant glioma. Recurrence of malignant glioma is often local, suggesting a role for a regional therapy. Gliadel wafers contain carmustine and are designed to release this agent over a 2- to 3-week period. Gliadel wafers are placed on the surface of the resected tumour beds in recurrent tumours and after initial resection. Data from phase i trials have demonstrated that Gliadel is safe and active in selected subgroups of patients with newly diagnosed and recurrent disease 1–3, and randomized data are now available. The Neuro-oncology Disease Site Group (dsg) felt that a systematic review of the evidence to provide an interpretation of the available clinical trials with respect to survival advantage, adverse events, and quality of life was warranted.

## 3. METHODS

### 3.1 Guideline Development

The present systematic review was originally completed in the context of developing an evidence-based series, including a clinical practice guideline, for Cancer Care Ontario’s Program in Evidence-Based Care (pebc), using the methodology of the practice guidelines development cycle 4. The evidence was selected and reviewed by members of the Neuro-oncology dsg and by methodologists. The pebc is editorially independent of Cancer Care Ontario and the Ontario Ministry of Health and Long-Term Care. Evidence-based series produced by the pebc undergo periodic review and evaluation of the literature, and new evidence is incorporated into the original reports as appropriate. The most recent versions of the reports can be found on the pebc Web site: www.cancercare.on.ca/index_practiceGuidelines.htm.

### 3.2 Literature Search Strategy

A systematic search of the medline (1990 to March 2006, week 3), embase (1990 to 2006, week 11), cancerlit (1990 to October 2002), and Cochrane Library (2006, Issue 1) databases was conducted. The terms “glioma” (Medical Subject Heading) and “brain neoplasms” were combined with the text words “Gliadel,” “carmustine,” and “bcnu.” In addition, the Physician Data Query clinical trials database (www.cancer.gov/clinical_trials/) and the proceedings of the 1997–2005 meetings of the American Society of Clinical Oncology were searched for reports of new or ongoing trials. Relevant articles and abstracts were selected and reviewed, and the reference lists from these sources were searched for additional trials.

### 3.3 Study Selection Criteria

Articles were selected for inclusion if they

- were fully published reports of rcts or systematic reviews of rcts comparing treatment with Gliadel wafers to placebo or alternative treatment in patients with malignant glioma. Prospective cohort studies were also included.
- included results regarding the safety or efficacy of Gliadel for patients with malignant glioma.

Articles were excluded from this systematic review of the evidence if they were

- letters and editorials.
- papers published in a language other than English.

## 4. RESULTS

### 4.1 Literature Search Results

In 2000, Engelhard published a review describing the role of interstitial bcnu chemotherapy in patients with malignant glioma 5. The Engelhard review included five studies, including two phase i studies^1^,^2^, one prospective cohort study with historical controls 6, and two rcts 7,8. Since the publication of the Engelhard review, one large rct evaluating the role of Gliadel in patients with malignant glioma has been published 9.

Three rcts 7–9 and one prospective cohort study with historical controls 6 were eligible for inclusion in this systematic review (Table I). A long-term follow-up study 10 for one of the rcts 9 was also included.

The rcts compared patients treated with Gliadel with patients treated with placebo, and all were supported by pharmaceutical funding. Two of the rcts studied patients with newly diagnosed malignant glioma 8,9. The third rct 7 and the prospective study^6^ investigated patients with recurrent malignant glioma. No studies comparing Gliadel with alternative treatment were identified.

### 4.2 Outcomes

#### 4.2.1 Efficacy

##### Newly Diagnosed Malignant Glioma

Two rcts compared Gliadel with placebo in patients with newly diagnosed malignant glioma 8,9. Westphal *et al.* 9 conducted a multicentre, double-blind phase iii rct that compared 120 patients in each study arm at the time of surgery. The sample size was specified in advance and based on a two-tailed log-rank test with an alpha level of 0.05 and a power of 0.90 to detect an 18% difference in 1-year survival between Gliadel and placebo (68% vs. 50%). The original course of the trial was 30 months, but a long-term follow-up study was later published, extending the follow-up to 56 months 10. Survival data for 58 patients who were known to be alive at the end of the original trial period were obtained retrospectively and were combined with data from the original study period for analysis. Over the 56-month period, only 1 patient was lost to follow-up.

Westphal *et al* 9,10 reported that overall survival at 1 year was 59.2% for the Gliadel patients and 49.2% for the placebo patients 10. Survival for the Gliadel and placebo groups was 15.8% and 8.3% respectively at 2 years and 9.2% and 1.7% respectively at 3 years. The difference between the survival curves was statistically significant [hazard ratio (hr): 0.73; 95% confidence interval (ci): 0.56 to 0.95; *p* = 0.018], with a 27% reduction in risk of death for patients receiving Gliadel as compared with those receiving placebo. Median survival was 13.8 months in the Gliadel arm and 11.6 months in the placebo arm (*p* = 0.017).

Because the high number of patients undergoing re-operation could have confounded the results (29% in the Gliadel arm and 25% in the placebo arm at 30 months), an analysis of the intent-to-treat population was performed, in which patients undergoing re-operation were censored at the time of surgery. That analysis was performed at the end of the 30-month study period, because no data for re-operation were available for the 58 patients who were followed long-term after that time point. In the resulting analysis, patients in the Gliadel group survived longer than did those in the placebo group (hr: 0.64; 95% ci: 0.45 to 0.92; *p* = 0.01), with a median survival of 64.1 weeks as compared with 49.4 weeks 9.

Westphal *et al.* 9,10 also analyzed their results in histologic subgroups. In the Gliadel arm, 101 patients had gbm, and in the placebo arm, 106 patients had gbm. For that subgroup, median survival was 13.1 months in the Gliadel arm and 11.4 months in the placebo arm. No significant difference in survival between the two gbm subgroups was detected (hr: 0.78; 95% ci: 0.595 to 1.03, *p* = 0.08) 10. When Westphal *et al.* corrected for the possible imbalance in prognostic factors (because the groups had not originally been randomized according to histologic subgroup), no significant survival advantage was detected for the patients with gbm in the Gliadel arm as compared with equivalent patients in the placebo arm (hr: 0.78; 95% ci: 0.58 to 1.05; *p* = 0.10). However, the trial was not designed to detect differences between histologic subgroups.

Valtonen *et al.* 8 reported the results of a small double-blind randomized trial in which 32 patients with newly diagnosed malignant glioma were randomized to receive either Gliadel or a placebo. Initially, the trial was designed to recruit 100 patients; however, because of difficulty obtaining Gliadel, the trial was terminated early. An imbalance was noted in the histologies in the two arms: 16 patients in the placebo arm had gbm (100%) as compared with 11 patients in the Gliadel arm (69%). Valtonen *et al.* 8 reported a statistically significant overall survival benefit (hr: 0.27; 95% ci: 0.11 to 0.68; *p* = 0.006) and increased median survival (58.1 weeks vs. 39.9 weeks, *p* = 0.012) in the Gliadel arm. A subgroup analysis of the 27 patients with grade iv tumours revealed a similar benefit for Gliadel in overall survival (hr: 0.27; 95% ci: 0.10 to 0.71; *p* = 0.008). Median survival for that subgroup of patients was 53.3 weeks in the treatment arm and 39.9 weeks in the placebo arm (*p* < 0.05). Those results need to be interpreted with caution because of the small number of patients and the small variances in prognostic factors, both of which could have significantly influenced outcome.

##### Recurrent Malignant Glioma

One rct examined the role of Gliadel in recurrent malignant glioma 7. Brem *et al.* compared 110 patients receiving Gliadel to 112 patients receiving a placebo. Each trial arm had a similar proportion of gbm patients: 65.5% in the Gliadel arm and 65.2% in the placebo arm. The analysis of overall treatment effect showed no significant benefit for Gliadel (hr: 0.83; 95% ci: 0.63 to 01.10; *p* = 0.19). However, once adjustment was made for the effects of prognostic factors, the overall treatment effect favoured Gliadel (hr: 0.67; 95% ci: 0.51 to 0.90; *p* = 0.006). The median survival was 31 weeks for the Gliadel arm and 23 weeks for the placebo arm. Overall patient survival at 6 months was 60% in the Gliadel arm and 47% in the placebo arm. That difference was nonsignificant (*p* = 0.061).

As in the rct by Westphal *et al.* 9, Brem *et al.* 7 compared histologic subgroups in the Gliadel and placebo arms. However, the results of these subgroup analyses need to be interpreted with caution, because the study was not designed to detect survival differences between subgroups. The authors reported that 6-month overall survival for gbm patients was 56% in the Gliadel arm and 36% in the placebo arm (*p* = 0.020). The estimated hr showed no significant difference between treatment arms (0.81, *p* = 0.22), but a benefit for Gliadel was observed after an adjustment for treatment group and prognostic factors (hr: 0.67; 95% ci: 0.48 to 0.95; *p* = 0.02).

One prospective cohort study with a historical control examined the role of Gliadel in patients with recurrent malignant glioma 6. In that study, 17 patients underwent surgery for recurrent malignant glioma and received Gliadel wafers. A cohort of 45 patients who had undergone surgery for recurrent malignant glioma during the same time period was retrospectively identified as a control group. The authors reported median survival from diagnosis as 58 weeks for the Gliadel group and 97 weeks for the control group. Although the authors reported no significant difference in prognostic factors between groups, a possible selection bias was suggested, because the patients offered Gliadel had no remaining treatment options. Patients in the control cohort received established adjuvant treatment. The potential for bias in nonrandomized studies with historical controls prevents any conclusions being drawn from the results of the study.

#### 4.2.2 Safety

Westphal *et al.* 9 reported that the number of deaths, adverse events, and laboratory abnormalities were high, as expected in this particular patient population. The Gliadel arm and the placebo arm both experienced similar adverse events. The most frequently reported adverse events among the patients receiving Gliadel were hemiplegia, convulsions, confusion, and brain edema. The most commonly reported adverse events among the patients in the placebo arm were convulsions, confusion, brain edema, and aphasia. The only difference between the groups in the Westphal *et al.* study 4 was that more patients in the Gliadel arm experienced intracranial hypertension (11 patients vs. 2 patients in the placebo arm, *p* = 0.019).

Valtonen *et al.* 8 reported results similar to those of Westphal *et al.* 9. They found that 12 patients in the treatment group and 9 patients in the placebo group reported adverse events. The most common adverse events among the patients in the Gliadel group were hemiparesis, convulsion, visual field defect, and aphasia.

Brem *et al.* 7 also found that both groups had similar occurrences of adverse events. They found that 2% of the patients in each group developed thrombocytopenia, and that 1% of the patients in the Gliadel group developed leukopenia. Brem *et al.* 7 also compared seizures between the groups. They found that 41 patients in the Gliadel group and 32 patients in the placebo group experienced seizures (*p* = 0.199). The overall incidence of serious intracranial infection was 2.2%, but this complication was more common in the Gliadel arm than in the placebo arm (3.6% and 0.89% respectively). This difference was statistically nonsignificant.

## 5. DISCUSSION

### 5.1 Newly Diagnosed Malignant Glioma

Two rcts compared the efficacy of Gliadel with placebo in patients with newly diagnosed gliomas 8,9. In the largest rct to date, patients who received Gliadel for newly diagnosed malignant glioma were reported to have experienced a 2-month improvement in median survival as compared with patients who received placebo (p = 0.017) 10. In addition, analysis of the survival curves revealed a significant 27% reduction in risk of mortality for patients who received Gliadel (*p* = 0.018). A survival advantage with Gliadel in patients with gbm was not detected, but the trial was not designed to make comparisons between histologic subgroups. Because the researchers in another randomized trial were unable to obtain sufficient Gliadel, that trial included only 32 patients newly diagnosed with malignant glioma instead of the anticipated 100 8. Although a survival benefit was reported for Gliadel in the overall patient population and in patients with gbm, no conclusions could be reached based on the small number of patients enrolled.

Both studies reported similar adverse events in the treatment and control arms. The most common adverse events associated with Gliadel were hemiplegia, convulsions, confusion, and brain edema. The most commonly reported adverse events among patients who received placebo were convulsions, confusion, brain edema, and aphasia. A significantly higher number of patients experienced intracranial hypertension in the Gliadel arm of the Westphal trial 9. Because neither trial included a comparison with systemic therapy, the possible contrast between the adverse event rates associated with interstitial chemotherapy wafers and the rates expected with systemic chemotherapy is unclear.

Given that the largest trial demonstrated a survival advantage in the Gliadel treatment arm, Gliadel may be considered an option in the subgroup of patients with newly diagnosed resectable malignant gliomas. However, the exact patient population (based on age, histology, performance status, and so on) that may benefit from Gliadel is unclear; further investigation is needed. In addition, no comparison has been performed between the efficacy of interstitial and systemic chemotherapy; clinicians should therefore review the latest evidence for the benefit of systemic chemotherapy in patients with newly diagnosed malignant glioma.

### 5.2 Recurrent Malignant Glioma

One rct compared the efficacy of Gliadel with that of placebo in patients with recurrent glioma 7. The overall result of that trial was negative, with no significant survival advantage seen in the primary analysis. However, a survival advantage for Gliadel was observed in the overall patient population and in patients with gbm after adjustment for prognostic factors. Given that no subgroups had been identified *a priori,* the results of the subgroup analysis of gbm patients in that trial should be interpreted with caution.

No survival advantage for Gliadel was detected in the cohort study with historical controls 2, but no conclusions can be reached because of the heterogeneity between patients and the potential for bias in studies of this nature.

The positive results of the rct 7 after adjustment for prognostic factors suggest that Gliadel may increase overall survival in some patients with recurrent resectable malignant glioma. Because such patients generally have a poor outlook, any treatment that has the potential for prolonging life without significant adverse events should be considered an option.

## 6. CONCLUSIONS

Evidence from rcts suggests a significant survival benefit for Gliadel as compared with placebo. Gliadel followed by standard radiotherapy is an option for selected patients with newly diagnosed malignant glioma where a near gross total resection is possible; however, most patients with malignant glioma will be ineligible for various reasons (non-resectable tumours or contact with the ventricular system). Similarly, Gliadel is an option in patients with surgically resectable recurrent malignant gliomas. The specific patient population (based on age, histology, performance status, and so on) that would benefit from Gliadel is unclear; further investigation is needed.

A direct comparison between Gliadel and systemic chemotherapy has not been undertaken; such a study would be helpful in defining the relative roles of this local therapy and systemic therapy. The current standard of care for patients with newly diagnosed gbm is radiotherapy with concurrent and adjuvant temozolomide. No evidence is currently available to support the sequential combination of Gliadel with temozolomide, and therefore a decision to use Gliadel with subsequent temozolomide should be made for patients individually, recognizing that little clinical experience with this combined treatment has accrued and that patients should be made aware of the possibility of increased toxicity. Clinical trials investigating the combination of Gliadel wafers with systemic therapy are required to further clarify this issue.

## Figures and Tables

**TABLE I:** Overview of studies included in this systematic review

Reference	Study design	Patients [*n* (% gbm)]	Experimental/ control	Additional treatment	Re-operation [n (%)]	Chemo- therapy [*n* (%)]	Median survival (weeks)	*p* Value	Mortality hazard ratio (95% ci)	*p* Value
Patients with newly diagnosed malignant glioma										
Valtonen *et al.* 1997 8	rct	16 (69) 16 (100)	3.85% bcnu Placebo	Standard rt after surgery	Subsequent operations allowed	nr	58.1 39.9	0.012	0.27 (0.11–0.68) b	0.006 b
Westphal *et al.* 2003 9,10	rct	120 (84) 120 (88)	3.85% bcnu Placebo	ebrt (2 weeks after surgery)	36 (30) a 30 (25) a	35 (29)^a^ 28 (23) a	59.8 50.3	0.017	0.73 (0.56–0.95)	0.018
Patients with recurrent malignant glioma										
Brem *et al.* 1995^7^	rct	110 (65)	3.85% bcnu	100% prior rt	No difference in number of prior surgeries (*p=*0.17)	52.7% prior chemo	31	nr	0.83 (0.63–1.10)	0.19
		112 (65)	Placebo			48.2% prior chemo	23		0.67 (0.51–0.90) c	0.006 c
Subach *et al.* 1999^6^	Cohort	17 (100)	bcnu	100% prior rt	76% prior craniotomy	88% prior chemo	58	nr	nr	<0.001 in favour of control
	Control	45 (100)	No treatment		71% prior craniotomy	96% prior chemo	97			

a No data available for patients in long-term follow-up study published in 2006 9. Data presented are for the original 30-month follow-up period. All patients receiving chemotherapy in this period also underwent re-operation. When the patients who underwent re-operation and chemotherapy were removed from the analysis at 30 months follow-up, median survival was 64.1 weeks in the bcnu group and 49.4 weeks in the control group (*p* = 0.02).

b See “Discussion” for results for patients with grade iv tumours only.

c After adjustment for prognostic factors.

gbm = glioblastoma multiforme; ci = confidence interval; rct = randomized controlled trial; bcnu = carmustine; ebrt = external-beam radio-therapy; rt = radiotherapy; nr = not reported; ns = nonsignificant.
